# Work–life balance and silent resignation among bank employees: the moderating roles of emotional intelligence and psychological empowerment

**DOI:** 10.3389/fpsyg.2026.1901929

**Published:** 2026-09-17

**Authors:** Hakan Uğurbulduk, İ. Efe Efeoğlu

**Affiliations:** Department of Business Administration, Adana Alparslan Türkeş Science and Technology University, Adana, Türkiye

**Keywords:** banking sector, emotional intelligence, psychological empowerment, silent resignation, work–life balance

## Abstract

**Introduction:**

Recent discussions on silent resignation have highlighted employees’ growing tendency to restrict their work contributions to formally required duties, raising concerns regarding employee well-being and sustainable work behavior. From an occupational psychology perspective, work-life balance constitutes an important psychological resource that may shape employees’ responses to workplace demands. Grounded in Social Exchange Theory, this study examines the relationship between work-life balance and silent resignation while investigating the moderating roles of emotional intelligence and psychological empowerment among bank employees.

**Methods:**

The study employs a quantitative, cross-sectional survey design with 518 white-collar employees across 69 provinces in Türkiye, analyzed using Partial Least Squares Structural Equation Modeling (PLS-SEM).

**Results:**

The findings indicate a strong negative association between perceived work-life balance and silent resignation. Furthermore, emotional intelligence acts as a significant individual-level boundary condition, such that the negative association between work-life balance and silent resignation becomes more pronounced at higher levels of emotional intelligence. In contrast, psychological empowerment does not significantly moderate this relationship, highlighting the potential contextual limits of empowerment in highly regulated and standardized work environments.

**Discussion:**

Practically, these findings suggest that organizations should prioritize work-life balance initiatives while considering employees’ emotional capabilities and ensuring that empowerment practices are supported by meaningful opportunities for autonomy and decision-making.

## 1 Introduction

Recent transformations in the workplace have reshaped the psychological and behavioral relationship between employees and organizations. Digitalization, increasing performance pressures, and the blurring of boundaries between professional and personal life have altered employee expectations, particularly in service-sector work. Consequently, work–life balance and the sustainability of employees’ work experiences have become increasingly important components of the employee–organization relationship. When these expectations are not met, employees may respond through forms of withdrawal that do not involve formally leaving the organization.

In the post-pandemic period, silent resignation, commonly referred to as “quiet quitting,” has received increasing scholarly attention as a form of employee withdrawal characterized by restricting work contributions to formally required duties while reducing discretionary and extra-role effort. Recent scholarship increasingly approaches silent resignation not simply as a motivational deficit, but as a context-dependent behavioral response associated with employees’ perceptions of their working conditions and employment relationships (Bordoloi et al., 2025; Harter, 2023; Magrizos et al., 2026; Mahand and Caldwell, 2023).

Work–life balance has emerged as an important work-related factor associated with silent resignation. When job demands persistently interfere with personal and family domains, employees may experience strain on their emotional and cognitive resources (Netemeyer et al., 1996). Recent empirical evidence further links work–life conditions with quiet quitting and related withdrawal tendencies (Nguyen and Vu, 2025; Paranamana and Kaluarachchige, 2025; Yisa et al., 2024). Rather than leaving their organizations entirely, employees experiencing unfavorable work–life conditions may respond by restricting discretionary contributions and aligning their effort more closely with formally required duties. However, employee responses to similar work–life conditions are unlikely to be uniform, suggesting that individual psychological characteristics and organizational perceptions may shape the strength of this relationship.

Emotional intelligence represents one potentially relevant individual-level characteristic because it influences how individuals perceive, interpret, and regulate emotional experiences associated with demanding work conditions. Recent research has linked emotional intelligence to employee well-being and withdrawal-related outcomes, including quiet quitting and burnout (Galanis et al., 2024; Rahmawati et al., 2025). Psychological empowerment represents a distinct but complementary psychological construct, reflecting employees’ perceptions of meaning, competence, self-determination, and impact at work. Although psychological empowerment is generally associated with favorable work outcomes, emerging research suggests that its consequences may depend on organizational and contextual conditions (Llorente-Alonso et al., 2024; Çetinkaya, 2025).

Banking work is characterized by intensive customer interaction, performance pressures, standardized processes, regulatory requirements, and relatively constrained decision-making structures. In addition, digitalization has expanded the technological connectivity of banking work, increasing the relevance of boundaries between work and non-work domains. These characteristics make banking an informative context for examining how work–life conditions are associated with withdrawal behavior and whether individual psychological characteristics and perceptions alter this relationship.

Although the literature on silent resignation has expanded rapidly, recent research remains dispersed across different individual, organizational, and contextual antecedents, with emerging reviews also highlighting sectoral variation and the need for more context-sensitive explanations of quiet quitting (Bordoloi et al., 2025; Magrizos et al., 2026; Maheshwari, 2026). Limited empirical attention has been devoted to the conditions under which work–life balance is associated with silent resignation, particularly when individual emotional capabilities and perceptions of psychological empowerment are considered simultaneously. This gap is especially relevant in highly regulated and standardized service settings, where employees may possess favorable psychological characteristics while operating within structural constraints that limit how these characteristics are expressed in workplace behavior.

To address this gap, the present study examines the relationship between work–life balance and silent resignation among white-collar bank employees and tests the moderating roles of emotional intelligence and psychological empowerment within the theoretical framework of Social Exchange Theory.

The study makes three contributions. First, it extends research on silent resignation by examining the phenomenon in relation to employees’ work–life conditions rather than treating it solely as an individual motivational deficit. Second, it examines emotional intelligence as an individual-level boundary condition that may shape the strength of the work–life balance–silent resignation relationship. Third, it investigates whether psychological empowerment conditions this relationship within a highly standardized and regulated work environment, thereby contributing to a more context-sensitive understanding of psychological empowerment.

## 2 Theoretical framework and hypothesis development

### 2.1 Theoretical background: social exchange theory

Social Exchange Theory (SET) provides the main theoretical framework for explaining silent resignation in this study. SET is based on the principle of reciprocity and suggests that relationships between employees and organizations develop through exchanges in which individuals evaluate the balance between their contributions and the benefits, support, and treatment they receive in return. When employees perceive the exchange relationship as fair and mutually beneficial, they are more likely to reciprocate through commitment, cooperation, and discretionary contributions. Conversely, when the exchange is perceived as imbalanced, employees may adjust their attitudes and behaviors by reducing contributions that extend beyond their formal role requirements.

Silent resignation can be interpreted within this reciprocal exchange process. Unlike formal turnover, silent resignation does not involve terminating the employment relationship. Instead, employees remain organizational members while restricting their contributions to formally required duties and reducing discretionary or extra-role effort. From an SET perspective, such behavioral adjustment may represent an attempt to recalibrate the employee–organization exchange when employees perceive that the demands or contributions expected from them exceed the benefits or support they receive in return (Atalay and Dağıstan, 2024; Mahand and Caldwell, 2023; Xueyun et al., 2023).

Recent research provides further support for examining quiet quitting through an exchange-based perspective. Gray et al. (2025) examined quiet quitting through psychological contract and social exchange perspectives and demonstrated that employees’ evaluations of psychological contract fulfillment and breach are relevant to understanding quiet quitting. More recent evidence further supports the relevance of exchange-based explanations of quiet quitting. Atiq et al. (2026), drawing partly on Social Exchange Theory, demonstrated that quiet quitting is embedded within a broader psychological architecture of employee disengagement involving work engagement, job satisfaction, and negative affect. This evidence reinforces the view that quiet quitting should not be understood solely as an individual motivational deficit, but may also reflect employees’ evaluations of their relationship with the organization. Recent conceptual developments further emphasize that quiet quitting encompasses heterogeneous behavioral patterns and motivations, while reduced discretionary or extra-role contribution remains a central feature of the phenomenon (Magrizos et al., 2026).

Accordingly, SET provides an appropriate theoretical basis for understanding silent resignation as a behavioral adjustment associated with perceived reciprocity in the employee–organization relationship. When employees perceive a favorable and balanced exchange, they may be more willing to contribute beyond minimum role requirements; when the exchange is perceived as unfavorable or imbalanced, restricting discretionary contributions may become one possible behavioral response. This perspective is particularly relevant to the present study because work–life balance represents an important aspect of employees’ evaluation of their employment conditions, while emotional intelligence and psychological empowerment may shape how employees interpret and respond to these conditions. The following sections therefore develop the hypothesized relationships between work–life balance, silent resignation, emotional intelligence, and psychological empowerment within this exchange-based framework.

### 2.2 Work–life balance and silent resignation

Work–life balance refers to the extent to which individuals can establish and maintain a satisfactory and sustainable equilibrium between the demands of their professional responsibilities and their personal or family life roles (Fisher-McAuley et al., 2003; Greenhaus et al., 2003). When workplace demands persistently interfere with the non-work domain, employees may experience work–family conflict and sustained strain across their work and personal lives (Brough et al., 2014; Netemeyer et al., 1996).

Recent empirical evidence increasingly links work–life conditions with silent resignation. Nguyen and Vu (2025), examining Generation Z employees in Vietnam, identified work–life balance as an important factor associated with quiet quitting. Similarly, Paranamana and Kaluarachchige (2025) found that higher work–life balance was associated with lower quiet quitting among factory employees, while job satisfaction partially mediated relationships involving work–life balance, burnout, and quiet quitting. At a broader level, Bordoloi et al. (2025) identified work-related and organizational conditions among the major antecedent categories emerging in the quiet quitting literature. Together, these findings suggest that work–life balance represents an important work-related condition associated with employees’ willingness to maintain or restrict discretionary contributions.

From a Social Exchange Theory perspective, work–life conditions can be interpreted as part of employees’ broader evaluation of their exchange relationship with the organization. When excessive workloads, extended working hours, or continuous connectivity persistently interfere with employees’ non-work lives without corresponding organizational support, employees may perceive the exchange relationship as increasingly imbalanced. Under such conditions, particularly when formal turnover is economically or practically difficult, employees may become less willing to sustain voluntary citizenship behaviors or extra-role contributions. Instead, they may recalibrate their contribution levels by withholding discretionary effort and restricting performance more closely to formally required duties (Anand et al., 2023; Nguyen and Vu, 2025; Paranamana and Kaluarachchige, 2025; Yisa et al., 2024).

Conversely, when employees perceive that their organization respects non-work boundaries and supports a sustainable relationship between work and personal life, the employment relationship may be evaluated more favorably, reducing the likelihood that employees restrict their discretionary contributions. This relationship may be especially relevant in demanding service environments such as banking, where intensive customer interaction, performance pressure, standardized procedures, and increasing technological connectivity can heighten the salience of work–life boundaries.

Based on the theoretical reasoning and empirical evidence presented above, the following hypothesis is proposed:

*H1*: The perception of work–life balance is negatively and significantly associated with silent resignation behavior.

### 2.3 The moderating role of emotional intelligence

Emotional intelligence refers to an individual’s capacity to perceive, understand, regulate, and utilize emotions effectively to guide cognitive processes and behavioral responses (Bar-On, 2006; Mayer et al., 2001; Petrides and Furnham, 2000). In workplace settings, emotional intelligence represents an important individual-level psychological capability that may shape how employees adapt to demanding environments, manage occupational stress, and respond to emotionally challenging work experiences (Deniz et al., 2013; Petrides et al., 2004). However, the specific contingency mechanisms through which emotional capabilities condition employee withdrawal behaviors, such as silent resignation, require closer theoretical scrutiny.

Grounded in Social Exchange Theory, employment relationships rely on norms of reciprocity whereby employees may adjust their discretionary contributions according to their evaluations of the employee–organization exchange (Miles, 2016). When work demands persistently interfere with employees’ personal lives, the exchange relationship may be perceived as increasingly unfavorable or imbalanced (Bordoloi et al., 2025). Nevertheless, employees’ appraisal of and behavioral responses to similar work–life conditions are unlikely to be uniform, because individuals differ in their capacity to perceive, understand, and regulate the emotional consequences of workplace experiences.

Recent empirical evidence supports the relevance of emotional intelligence to both work–life experiences and withdrawal-related outcomes. Galanis et al. (2024) found that higher emotional intelligence was associated with lower quiet quitting, turnover intention, and job burnout among nurses, directly linking emotional intelligence with withdrawal-related outcomes. Rahmawati et al. (2025) further identified emotional intelligence as a mediating mechanism in the relationship between work–family conflict and job performance, suggesting that emotional capabilities may influence how work–family experiences are translated into employee outcomes. Importantly, recent research has also begun to examine emotional intelligence as a boundary condition in relationships involving work–life balance. Park and Kim (2025), for example, incorporated emotional intelligence as a moderator within a model linking perceived work–life balance, workplace compassion, and organizational commitment. Together, these studies indicate that emotional intelligence may function not only as an independent psychological capability but also as a contingency factor shaping how employees respond to their work–life conditions.

From an SET perspective, this contingency role is theoretically relevant because employees with different levels of emotional intelligence may appraise and regulate the emotional consequences of favorable and unfavorable exchange conditions differently. Emotional intelligence should therefore not necessarily be conceptualized as an automatic protective buffer that uniformly reduces withdrawal. Rather, it may alter the extent to which employees translate their perceptions of work–life conditions into decisions about discretionary contribution. In favorable work–life conditions, employees with greater emotional capabilities may respond more positively to a balanced exchange relationship; under unfavorable conditions, the same capabilities may facilitate greater recognition and regulation of the emotional consequences associated with perceived imbalance. Thus, the strength of the relationship between work–life balance and silent resignation may vary according to employees’ level of emotional intelligence.

Accordingly, emotional intelligence can be understood as an individual-level boundary condition that moderates the relationship between work–life balance and silent resignation. Therefore, the following hypothesis is proposed:

*H2*: Emotional intelligence moderates the relationship between the perception of work–life balance and silent resignation behavior.

### 2.4 The moderating role of psychological empowerment

Psychological empowerment represents an intrinsic task motivation manifested in four cognitive dimensions: meaning, competence, self-determination, and impact (Spreitzer, 1995; Thomas and Velthouse, 1990). Meaning reflects the perceived value of work goals, competence refers to an individual’s belief in their capability to perform work activities, self-determination reflects perceived autonomy in initiating and regulating work behaviors, and impact concerns the extent to which individuals perceive that they can influence organizational outcomes. In organizational research, psychological empowerment has generally been associated with favorable employee outcomes, including greater job satisfaction and organizational commitment and lower turnover intentions (Çetinkaya, 2025; Karakaş and Serçek, 2014; Llorente-Alonso et al., 2024).

Nevertheless, the implications of psychological empowerment may depend on the organizational context in which employees exercise meaning, competence, self-determination, and impact. In highly standardized and regulated work environments such as commercial banking, employees may experience psychological empowerment while simultaneously operating within formal procedures, compliance requirements, performance controls, and relatively restricted decision boundaries. This distinction between perceived psychological empowerment and the structural opportunities available to exercise discretion is theoretically important because employees’ perceptions of autonomy and impact may not always translate into equivalent control over workload, scheduling, or work–life boundaries.

Existing empirical evidence provides a basis for considering psychological empowerment in relation to withdrawal behavior. Lu et al. (2023) demonstrated that psychological empowerment was relevant to quiet quitting and moderated relationships involving employee well-being and job burnout, suggesting that empowerment can operate as a contingency factor in withdrawal-related processes. More broadly, the meta-analysis by Llorente-Alonso et al. (2024) indicates that the relationships between psychological empowerment and employee outcomes are contingent on individual and contextual characteristics. However, existing research has primarily examined psychological empowerment in relation to work-domain attitudes and experiences, while considerably less is known about whether it changes the extent to which work–life conditions are associated with silent resignation.

From a Social Exchange Theory perspective, psychological empowerment may influence how employees evaluate and respond to the quality of their exchange relationship with the organization. Employees who experience greater meaning, competence, self-determination, and impact may perceive greater agency in responding to workplace demands and determining their discretionary contributions. However, the extent to which this perceived agency shapes behavioral responses may also depend on the structural opportunities available for exercising autonomy and influence. Thus, psychological empowerment may alter the strength of the relationship between employees’ work–life conditions and their decision to restrict discretionary contributions, although the nature and magnitude of this contingency remain an empirical question (Figure 1).

**Figure 1:**
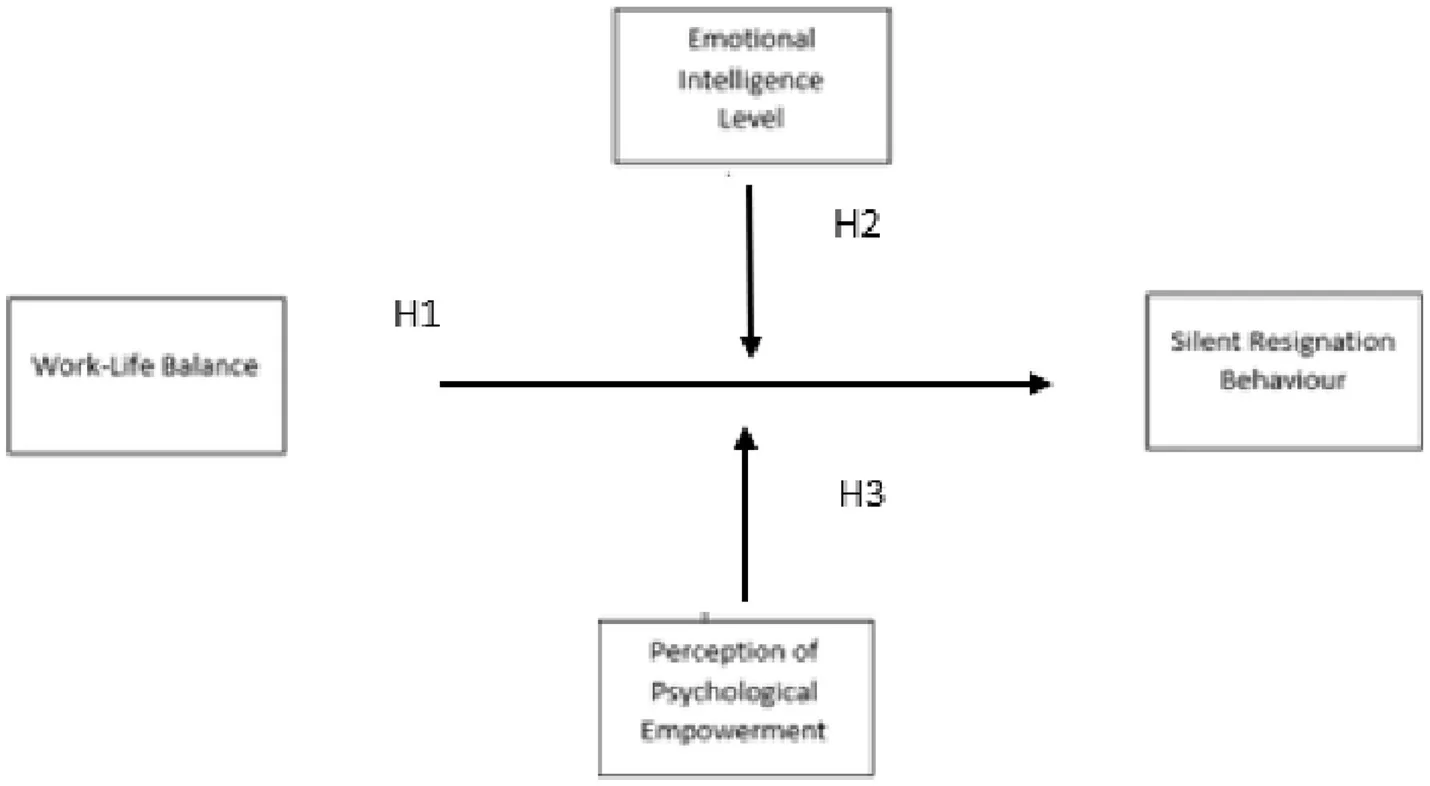
Conceptual research model and hypothesized relationships. Original figure created by the author(s).

Accordingly, the following hypothesis is proposed:

*H3*: Psychological empowerment moderates the relationship between the perception of work–life balance and silent resignation behavior.

## 3 Method

### 3.1 Research design

This study employs a quantitative, cross-sectional research design. Cross-sectional survey designs are widely used in organizational behavior and human resource research to examine relationships among variables at a specific point in time (Creswell et al., 2017). The primary objective of this study is to examine the relationship between work–life balance and silent resignation and to test the moderating roles of emotional intelligence and psychological empowerment in this relationship. Accordingly, the research model was structured to evaluate the proposed relationships and interaction effects among the study variables.

Given that the study examines multiple perception-based psychological and behavioral constructs and includes moderating relationships, Partial Least Squares Structural Equation Modeling (PLS-SEM) was employed to estimate the proposed model.

To minimize the risk of common method bias, procedural remedies were implemented during data collection. Anonymity was ensured, and participants were informed that there were no right or wrong answers. In addition, collinearity diagnostics were examined as an additional diagnostic for potential common method bias. The maximum inner VIF values ranged from 1.000 to 1.281, remaining well below the conservative threshold of 3.30. These results suggest that common method bias was unlikely to pose a serious threat to the interpretation of the findings. Nevertheless, because all variables were collected through self-report measures at a single point in time, the possibility of common method bias cannot be completely ruled out. Ethical approval for the study was obtained from the Ethics Committee under approval number E-64501449-050.04-113322.

### 3.2 Population and sample

The study population consists of white-collar employees working at branches of a private bank operating throughout Türkiye. The banking sector provides a relevant context for examining work–life balance and silent resignation because banking work is characterized by high workloads, intensive performance monitoring, standardized processes, and relatively limited time flexibility.

As of March 2025, a total of 188,971 people were employed in banks across Türkiye (Türkiye Bankalar Birliği, 2025). A minimum sample size of 384 was estimated for this population at a 95% confidence level using a sample-size calculator. The final sample consisted of 518 respondents and therefore exceeded this minimum requirement. Participants were recruited from bank branches across 69 provinces, and participation was voluntary. The sample comprised 61 branch managers (11.8%), 176 supervisors (34.0%), and 281 clerks (54.2%), representing different hierarchical levels within bank branches.

Data were collected through an online SurveyMonkey questionnaire between January and April 2025. Participation was voluntary, and anonymity and confidentiality were ensured throughout the data collection process. The survey was distributed to 620 bank employees, of whom 518 completed the questionnaire, corresponding to a response rate of 83.5%. A pilot test was conducted with 22 participants in January 2025 prior to the main data collection (Table 1).

**Table 1:** Demographic characteristics of the participants.

Demographic variable	Sub-group	Frequency (n)	Percentage (%)
Gender	Female	307	59.3
	Male	211	40.7
Marital status	Married	381	73.5
	Single	137	26.5
Age	20–30	92	17.7
	31–40	130	25.2
	41 and older	296	57.1
Tenure	<= 5 years	117	22.6
	6–10 years	55	10.7
	11–15 years	117	22.6
	16–20 years	159	30.6
	> = 20 years	70	13.5
Job title	Branch manager	61	11.8
	Supervisor	176	34.0
	Clerk	281	54.2

### 3.3 Data collection tool and measurement variables

The English sample items reported below are translations of the Turkish items administered to participants. A structured questionnaire was used as the data collection instrument, consisting of validated measurement scales alongside demographic questions. The study variables were measured using 5-point Likert-type response formats (1 = *Strongly Disagree* to 5 = *Strongly Agree*), except for emotional intelligence, which was assessed on a 7-point Likert scale (1 = *Strongly Disagree* to 7 = *Strongly Agree*).

- *Work–Life Balance (Work-to-Family Conflict)*: Work–life balance was assessed using the five-item work-to-family conflict subscale developed by Netemeyer et al. (1996) and adapted to Turkish by Yavuz and Doğan (2019) and Kaçik and Aykan (2022). In line with the focus of the present study on the interference of occupational demands with employees’ non-work lives, only the work-to-family dimension was used. Items were reverse-scored so that higher scores represented more favorable work–life balance. A sample item is: “My work responsibilities negatively affect my family and home life”.
- *Silent resignation*: Silent resignation was assessed using the seven-item unidimensional scale developed by Anand et al. (2023) and validated in Turkish by Yıldız et al. (2024). The scale reflects employees’ tendency to restrict their contributions to formally required duties and reduce discretionary effort. Sample items include: “I do the minimum amount of work required to continue in my position,” and “Unless there is extra compensation, I usually avoid doing additional work”. Following evaluation of the measurement model, items with outer loadings below 0.40 were removed, resulting in four retained items in the final measurement model.
- *Emotional intelligence*: Trait emotional intelligence was measured using the 20-item Trait Emotional Intelligence Questionnaire–Short Form (TEIQue-SF), developed by Petrides and Furnham (2000, 2001) and adapted to Turkish by Deniz et al. (2013). The Turkish version comprises four interrelated dimensions: well-being, self-control, emotionality, and sociability. Sample items include: “I generally find it difficult to regulate my emotions,” and “In general, I can cope with stress.” Responses were recorded on a 7-point Likert scale, and negatively worded items were reverse-coded in accordance with the scoring procedure.
- *Psychological empowerment*: Psychological empowerment was measured using Spreitzer’s (1995) 12-item multidimensional scale, adapted to Turkish by Sürgevil et al. (2013). The instrument comprises four dimensions with three items each: meaning, competence, self-determination, and impact. Representative items include: meaning— “The work I do is meaningful to me”; competence— “I am confident in my ability to do my job”; self-determination— “I have significant autonomy in determining how I do my job”; and impact— “My impact on what happens in my department is substantial.” Items were rated on a 5-point Likert scale.

### 3.4 Data analysis and the rationale for choice of PLS-SEM

PLS-SEM was used to estimate the proposed research model. The method was selected primarily because the study focuses on explaining variance in silent resignation and testing multiple interaction effects within a model containing several latent constructs (Sönmez Çakır, 2020; Yıldız, 2024; Yılmaz et al., 2024). PLS-SEM provides a flexible framework for estimating such models and testing moderation through interaction terms. In addition, because perception-based psychological constructs may exhibit departures from multivariate normality, the distributional flexibility of PLS-SEM was considered appropriate for the characteristics of the present data. Accordingly, the choice of PLS-SEM was aligned with the explanatory and moderation-oriented objectives of the study rather than being based solely on model complexity or distributional considerations.

The measurement model was evaluated before the structural model and hypothesis testing. Internal consistency reliability, convergent validity, and discriminant validity were assessed for the measurement model. The structural model was subsequently evaluated using path coefficients, explained variance (R^2^), effect sizes (f^2^), collinearity diagnostics, and the statistical significance of the hypothesized relationships and interaction terms. IBM SPSS Statistics was used for descriptive statistics, distributional assessments, silent resignation levels, and subgroup comparisons, whereas SmartPLS 4 was used to evaluate the measurement and structural models and to test the moderating effects. Statistical significance of the path coefficients and interaction terms was assessed using non-parametric bootstrapping with 10,000 subsamples.

## 4 Findings

The mean silent resignation score for the overall sample was 2.63. The distribution of silent resignation scores showed a skewness value of 0.268 and a kurtosis value of −0.089, indicating no substantial departure from univariate normality. Significant differences were observed across occupational groups (ANOVA, *p* < 0.001). *Post hoc* comparisons indicated that branch managers reported significantly lower levels of silent resignation (*M* = 2.12) than both supervisors (*M* = 2.70) and clerks (*M* = 2.70; *p* < 0.001). Participants also reported relatively high levels of emotional intelligence (*M* = 5.13) and psychological empowerment (*M* = 4.03).

### 4.1 Evaluation of the measurement model

Before proceeding to test the research model, the reliability and validity criteria of the measurement model were evaluated. The measurement model was examined in terms of internal consistency, convergent validity, and discriminant validity.

Items of the silent resignation construct with outer loadings below 0.40 were excluded, and the analysis proceeded with the four retained items (Hair et al., 2014; Sönmez Çakır, 2020; Yıldız, 2024; Yılmaz et al., 2024).

Internal consistency reliability was assessed using Cronbach’s alpha and composite reliability (CR). The reliability coefficients were above acceptable thresholds for all constructs, including silent resignation (0.80), work–life balance (0.92), emotional intelligence (0.96), and psychological empowerment (0.93), indicating satisfactory internal consistency.

Convergent validity was assessed using outer loadings and average variance extracted (AVE). The AVE values were 0.508 for silent resignation, 0.699 for work–life balance, 0.615 for emotional intelligence, and 0.514 for psychological empowerment. These values met the recommended threshold of 0.50, supporting the convergent validity of the constructs.

Discriminant validity was assessed to test whether the constructs were conceptually distinct from one another. The results obtained in this context indicate that each variable can be distinguished from the others. Findings regarding the measurement model indicate that the necessary conditions for proceeding to structural equation modeling have been met. For all variables, cross-loadings were higher than those of other variables in the same row; according to the Fornell-Larcker criterion, the relevant variable had the highest value within the same row and column; and the HTMT was below 0.90 (Tables 2, 3).

**Table 2:** Measurement model reliability and convergent validity.

Latent construct	Final items	Factor loadings	Cronbach’s 𝛼	Composite reliability (CR)	Average variance extracted (AVE)
Work–life balance	5 Items	0.702- 0.930	0.920	0.920	0.699
Silent resignation	4 Items	0.649- 0.825	0.802	0.803	0.508
Emotional intelligence	20 Items	0.585- 0.892	0.968	0.969	0.615
Psychological empowerment	12 Items	0.512- 0.905	0.932	0.925	0.514

**Table 3:** Comparison of measurement diagnostics, collinearity, explanatory power, and effect sizes across models.

Assessment	Indicator/metric	Direct model	EI moderation model	PE moderation model	Recommended criterion/benchmark
Discriminant validity	HTMT (maximum)	0.617	0.484	0.474	< 0.90
Discriminant Validity	Fornell– Larcker Criterion	Satisfied	Satisfied	Satisfied	√AVE > inter- construct correlations
Collinearity	Inner VIF (maximum)	1.000	1.281	1.068	< 3.30
Explanatory power	*R*^2^ – Silent Resignation	0.383	0.445	0.504	—
Predictive relevance	*Q*^2^_predict	0.282	—	—	> 0 (Predictive relevance)
Effect size	*f*^2^ – WLB → Silent Resignation	0.620	0.350	0.526	0.02/0.15/0.35 (Small/Medium/Large)
Effect size	*f*^2^ – Moderator → Silent Resignation	—	0.112 (EI)	0.242 (PE)	0.02, 0.15, 0.35 (Small/Medium/ Large)
Effect size	*f*^2^ – Interaction Term (WLB × Moderator)	—	0.015	0.004	0.02/0.15/0.35 (Small/Medium/Large)

### 4.2 Evaluation of the structural model

Following the validation of the measurement model, the structural model was evaluated to test the research hypotheses. Work–life balance was specified as the independent variable and silent resignation as the dependent variable, while emotional intelligence and psychological empowerment were examined as moderating variables. The statistical significance of the path coefficients and interaction effects was assessed using bootstrapping with 10,000 subsamples.

The structural model indicated a strong and statistically significant negative association between work–life balance and silent resignation [*β* = −0.619, *t* = 16.378, *p* < 0.001, 95% CI (−0.687, −0.538)]. Thus, higher perceived work–life balance was associated with lower levels of silent resignation, supporting Hypothesis 1.

The direct model explained 38.3% of the variance in silent resignation (*R*^2^ = 0.383). The effect size associated with work–life balance was large (*f*^2^ = 0.620), indicating that work–life balance made a substantial contribution to the explained variance in silent resignation. The PLSpredict analysis yielded a Q^2^_predict value of 0.282 for silent resignation. As this value was greater than zero, the model demonstrated out-of-sample predictive relevance for the target construct.

### 4.3 The moderating effect of emotional intelligence

Under the second hypothesis of the study, the moderating role of emotional intelligence in the relationship between work–life balance and silent resignation was tested. The interaction term between emotional intelligence and work–life balance was statistically significant [*β* = −0.089, *t* = 2.135, *p* = 0.033, 95% bias-corrected CI (−0.171, −0.006)]. Because the confidence interval does not include zero, emotional intelligence significantly moderates the relationship between work–life balance and silent resignation. Therefore, Hypothesis H2 is supported.

With the inclusion of emotional intelligence and its interaction term, the R^2^ value for silent resignation increased from 0.383 to 0.445, indicating an increase in the model’s explanatory power.

The negative interaction coefficient indicates that the negative association between work–life balance and silent resignation becomes stronger at higher levels of emotional intelligence. In other words, the association between higher work–life balance and lower silent resignation is more pronounced among employees with higher emotional intelligence.

### 4.4 The moderating effect of psychological empowerment

Under the third hypothesis of the study, the moderating role of psychological empowerment in the relationship between work–life balance and silent resignation was tested. The interaction term between psychological empowerment and work–life balance was not statistically significant [*β* = 0.035, *t* = 0.724, *p* = 0.469, 95% CI (−0.066, 0.118)]. Because the confidence interval includes zero, psychological empowerment does not significantly moderate the relationship between work–life balance and silent resignation. Therefore, Hypothesis H3 is not supported.

This finding suggests that psychological empowerment did not function as a significant moderating mechanism in the present sample. In other words, the relationship between work–life balance and silent resignation did not vary significantly according to employees’ levels of psychological empowerment.

This result may reflect the contextual characteristics of the banking sector. In highly regulated and standardized work environments with relatively constrained decision-making autonomy, perceived psychological empowerment may be insufficient to alter the relationship between work–life balance and silent resignation. This contextual interpretation is examined further in the Discussion section (Table 4).

**Table 4:** Structural model results and hypotheses testing outcomes.

Hypothesis and structural path	Path Coeff. (β)	t-value	*p*-value	95% confidence interval	Outcome
H1: WLB → Silent Resignation	−0.619	16.378	<0.001	[−0.687, −0.538]	Supported
H2: WLB × EI → Silent Resignation	−0.089	2.135	0.033	[−0.171, −0.006]	Supported
H3: WLB × PE → Silent Resignation	0.035	0.724	0.469	[−0.066, 0.118]	Not Supported

### 4.5 Overall evaluation of the findings

Overall, the findings indicate that work–life balance is strongly and negatively associated with silent resignation and that the strength of this association varies according to employees’ emotional intelligence. Specifically, the negative association between work–life balance and silent resignation becomes more pronounced at higher levels of emotional intelligence. In contrast, psychological empowerment did not significantly moderate this relationship. Taken together, the findings suggest that silent resignation may be understood partly in relation to employees’ perceived work–life conditions and that individual psychological characteristics do not uniformly condition this association.

## 5 Discussion

This study examined the relationship between work–life balance and silent resignation among bank employees and tested the moderating roles of emotional intelligence and psychological empowerment. The findings indicate that silent resignation is strongly associated with employees’ perceived work–life conditions and that this association varies according to emotional intelligence. From a Social Exchange Theory perspective, these findings are consistent with interpreting silent resignation as a form of behavioral recalibration within the employee–organization exchange rather than solely as an expression of individual reluctance.

### 5.1 Work–life balance and silent resignation: a behavioral recalibration mechanism

The structural model results indicate a strong, negative, and statistically significant association between perceived work–life balance and silent resignation, supporting Hypothesis 1. Specifically, employees reporting more favorable work–life balance also reported lower levels of silent resignation. Conversely, greater work-to-family interference was associated with a stronger tendency to restrict contributions to formally required duties.

Grounded in Social Exchange Theory, employment relationships are underpinned by reciprocal exchanges in which employees evaluate their investments of time, effort, and emotional energy in relation to the inducements, support, and working conditions provided by the organization (Miles, 2016). When occupational demands persistently interfere with employees’ personal and family lives without corresponding organizational support, the exchange relationship may increasingly be perceived as imbalanced. In high-pressure operational contexts such as banking, where employees work within standardized procedures and relatively constrained employment arrangements, silent resignation may represent a comparatively low-risk form of behavioral recalibration. Rather than formally leaving the organization, employees may respond to a perceived imbalance by restricting discretionary contributions and aligning their effort more closely with formally required duties.

This interpretation is consistent with recent empirical evidence linking work–life conflict, excessive job demands, and adverse work conditions with quiet quitting. Nguyen and Vu (2025) identified work–life balance as an important correlate of quiet quitting among Generation Z employees, while Paranamana and Kaluarachchige (2025) reported a negative relationship between work–life balance and quiet quitting among manufacturing employees. More recently, Máynez-Guaderrama and Cavazos-Arroyo (2025) showed that work–family and work–leisure conflict were associated with emotional exhaustion, which in turn was linked to quiet quitting among employees in the Mexican export manufacturing sector. Recent 2026 evidence provides both convergent and contrasting findings. Akdag et al. (2026) reported that more favorable work-to-life spillover was associated with lower quiet quitting among physicians, broadly aligning with the negative association observed in the present study. In contrast, Turan Aydın et al. (2026) did not identify a significant relationship between work–life balance and quiet quitting among construction-sector employees. Taken together, these differing findings suggest that the strength of the association between work–life conditions and quiet quitting may vary across occupational and organizational contexts. This interpretation is also consistent with broader reviews identifying work-related, organizational, and sector-specific conditions as relevant to understanding quiet quitting (Bordoloi et al., 2025; Maheshwari, 2026; Yisa et al., 2024).

The present study extends this evidence to a highly regulated banking context. Whereas previous research has examined Generation Z employees, manufacturing workers, and broader occupational samples, the current findings indicate that work–life conditions remain strongly associated with silent resignation even in a sector characterized by standardized procedures, performance pressure, intensive customer interaction, and relatively limited time flexibility. This suggests that silent resignation should not be understood solely as an individual motivation problem; rather, it may also reflect how employees evaluate the sustainability and reciprocity of their employment relationship.

The subgroup findings provide an additional observation consistent with this exchange-based interpretation. Branch managers reported lower silent resignation (*M* = 2.12) than both supervisors and clerks (*M* = 2.70 for each group). One possible interpretation from an SET perspective is that managerial positions may involve greater material and non-material organizational inducements, including compensation, benefits, status, decision-making authority, and perceived influence, which may contribute to a more favorable evaluation of the employee–organization exchange. However, because these specific inducements were not directly measured, this explanation should be regarded as theoretically plausible rather than as an empirically tested mechanism.

### 5.2 Emotional intelligence as a boundary condition in the work–life balance–silent resignation relationship

A key theoretical and empirical contribution of this study is the finding that emotional intelligence significantly moderates the relationship between work–life balance and silent resignation, supporting Hypothesis 2. The negative interaction coefficient indicates that the negative association between favorable work–life balance and silent resignation is more pronounced among employees with higher emotional intelligence. Thus, employees with higher emotional intelligence appear to show greater behavioral differentiation according to the quality of their perceived work–life conditions.

This finding provides a nuanced refinement to perspectives that conceptualize emotional intelligence primarily as a protective psychological resource. Individuals with higher trait emotional intelligence generally possess stronger capacities for emotional awareness, appraisal, and regulation (Bar-On, 2006; Deniz et al., 2013; Mayer et al., 2001). From a Social Exchange Theory perspective, these capabilities may increase employees’ sensitivity to the quality of the employee–organization exchange. Rather than uniformly insulating employees from unfavorable work conditions, emotional intelligence may influence how distinctly employees evaluate and respond to favorable versus unfavorable work–life conditions.

When employees perceive favorable work–life conditions, those with higher emotional intelligence may be particularly responsive to this positive aspect of the employment exchange and correspondingly less inclined to restrict discretionary contributions. Conversely, when work–life conditions are unfavorable, greater emotional awareness may make the imbalance between organizational demands and non-work needs more salient. Under such conditions, emotional intelligence may shape how employees adjust their discretionary contributions rather than simply preventing withdrawal. This interpretation is consistent with research showing that emotional intelligence plays an important role in how work–family experiences are translated into employee outcomes (Rahmawati et al., 2025).

Accordingly, emotional intelligence appears to function as an evaluative boundary condition rather than as a universally protective buffer. The present findings extend the emotional intelligence literature by suggesting that its organizational implications depend partly on the conditions employees are evaluating. In the banking context examined here, higher emotional intelligence was associated with a stronger differentiation in silent resignation across levels of perceived work–life balance. This provides a more conditional understanding of emotional intelligence and suggests that emotionally capable employees may be especially responsive to the reciprocity they perceive in their employment relationship.

### 5.3 The contextual limits of psychological empowerment

Contrary to Hypothesis 3, psychological empowerment did not significantly moderate the relationship between work–life balance and silent resignation. The non-significant interaction indicates that, within the present sample, the strength of the association between work–life balance and silent resignation did not vary significantly according to employees’ levels of psychological empowerment.

This finding is theoretically meaningful because it points to potential contextual boundaries in the operation of psychological empowerment. Psychological empowerment reflects employees’ perceptions of meaning, competence, self-determination, and impact. However, these psychological perceptions do not necessarily imply equivalent control over the work conditions that affect employees’ non-work lives. In commercial banking, standardized procedures, compliance requirements, centralized decision-making, and continuous performance monitoring may limit employees’ practical discretion over workloads, deadlines, or working hours even when they experience relatively high levels of psychological empowerment.

From this perspective, psychological empowerment may represent a favorable psychological experience without necessarily changing the relationship between work–life conditions and silent resignation. Employees may feel competent, meaningful, or influential in their work while still having limited opportunities to alter the structural demands that interfere with their personal and family lives. Consequently, perceived empowerment may not be sufficient to change how strongly work–life balance is associated with employees’ tendency to restrict discretionary contributions.

This interpretation is consistent with research suggesting that the consequences of psychological empowerment depend on contextual conditions rather than operating uniformly across all organizational settings (Llorente-Alonso et al., 2024). It is also compatible with Lu et al. (2023), who demonstrated that psychological empowerment can function as a contingency factor in some withdrawal-related processes. More recent evidence further reinforces the relevance of psychological empowerment to quiet quitting. Çavuşoğlu (2026), examining employees in the tourism sector, reported a significant relationship between psychological empowerment and quiet quitting. However, this evidence concerns the relationship between empowerment and quiet quitting rather than whether empowerment moderates the association between work–life balance and quiet quitting. The present non-significant interaction therefore adds a distinct contingency perspective, suggesting that psychological empowerment may be relevant to quiet quitting without necessarily altering the strength of the work–life balance–silent resignation relationship.

Accordingly, the non-significant moderating effect observed in the present study should not be interpreted as evidence that psychological empowerment is unimportant for employee outcomes. Rather, it suggests that its capacity to alter the association between work–life balance and silent resignation may depend on whether employees’ perceived empowerment is accompanied by meaningful opportunities to exercise autonomy over the specific work demands that shape their work–life boundaries. In the highly regulated banking context examined here, such structural constraints may help explain why psychological empowerment did not emerge as a significant boundary condition.

### 5.4 Theoretical contributions

This study advances organizational psychology and employee withdrawal scholarship on three distinct but interrelated theoretical levels. First, by framing silent resignation through Social Exchange Theory (SET), the study contributes to a more nuanced understanding of contemporary employee withdrawal behaviors. Rather than approaching silent resignation exclusively as an individual motivational deficit or an isolated performance problem, the findings suggest that restricting discretionary contributions may also be understood as a form of behavioral recalibration associated with employees’ evaluations of their work–life conditions. This perspective extends SET to a form of covert withdrawal in which employees remain within the organization while adjusting the extent of their discretionary contributions in response to the perceived quality of the employment exchange.

Second, the study refines the theoretical role of emotional intelligence by showing that trait emotional intelligence does not operate merely as a protective buffer against unfavorable work conditions. Instead, its significant moderating role positions emotional intelligence as an individual-level boundary condition that shapes the strength of the association between work–life balance and silent resignation. Employees with higher emotional intelligence exhibited greater differentiation in silent resignation across levels of perceived work–life balance. From an SET perspective, this pattern suggests that emotional capabilities may influence employees’ sensitivity and responsiveness to the quality of the employment exchange rather than uniformly reducing withdrawal under unfavorable conditions.

Third, the non-significant moderating role of psychological empowerment identifies a potential contextual boundary to assumptions regarding the beneficial role of empowerment. In highly standardized and regulated environments such as commercial banking, employees’ perceptions of meaning, competence, self-determination, and impact may not necessarily translate into equivalent operational control over workloads, working hours, or work–life boundaries. The findings therefore suggest that psychological empowerment should not automatically be expected to alter the relationship between unfavorable work–life conditions and withdrawal-related behavior. More broadly, they highlight the importance of considering whether perceived empowerment is accompanied by meaningful opportunities to exercise control over the specific work conditions relevant to employees’ work–life experiences.

Taken together, these contributions extend SET by demonstrating that the association between work–life conditions and covert withdrawal is conditional rather than uniform. The findings suggest that this relationship may vary according to individual emotional capabilities, while the relevance of other psychological resources may depend on the structural context in which they are exercised. This integrated perspective moves the silent resignation literature beyond explanations based solely on either individual characteristics or organizational conditions and emphasizes the interplay among exchange evaluations, individual differences, and contextual constraints.

## 6 Conclusions, managerial implications, limitations, and future research

### 6.1 Conclusion

This study examined the relationship between work–life balance and silent resignation among commercial bank employees while investigating the moderating roles of emotional intelligence and psychological empowerment through the theoretical lens of Social Exchange Theory (SET). The findings indicate a strong negative association between work–life balance and silent resignation, suggesting that silent resignation should not be understood solely as an individual motivational phenomenon but may also be considered in relation to employees’ work–life conditions within the broader employee–organization exchange.

Furthermore, the study identifies emotional intelligence as an important individual-level boundary condition that amplifies behavioral differentiation: the negative association between favorable work–life balance and silent resignation becomes more pronounced among employees with higher emotional intelligence. In contrast, psychological empowerment does not significantly moderate this relationship, suggesting that perceived empowerment may be insufficient to alter the association between work–life conditions and silent resignation within highly regulated and standardized operational environments.

### 6.2 Management implications

The findings offer actionable insights for executive leadership and human resource practitioners, particularly in banking and other highly regulated financial service environments. The strong negative association between work–life balance and silent resignation highlights the importance of protecting employees’ work–life boundaries. Rather than relying primarily on individual-level interventions to address withdrawal-related behavior, organizations should review workload distribution, after-hours task demands, and expectations of continuous digital availability. Clear digital disconnection practices, sustainable workload allocation, and greater managerial attention to employees’ non-work boundaries may help create work conditions associated with lower levels of silent resignation.

The moderating role of emotional intelligence further indicates that employees with higher emotional intelligence show greater differentiation in silent resignation across different levels of perceived work–life balance. Organizations may support emotional skills such as emotional awareness, regulation, and interpersonal sensitivity; however, such initiatives should not be treated as substitutes for favorable work–life conditions. Emotionally capable employees may be particularly responsive to the quality of their work–life experiences, reinforcing the need to combine individual development initiatives with sustainable organizational practices.

The non-significant moderating role of psychological empowerment also has important managerial implications. Perceived empowerment alone may be insufficient to alter the relationship between work–life conditions and silent resignation, particularly in standardized and compliance-intensive environments. Organizations should therefore seek to complement psychological empowerment with meaningful opportunities for employee influence where operational and regulatory requirements permit. Practical initiatives may include participation in workload planning, realistic goal setting, task prioritization, scheduling flexibility where feasible, and participative problem-solving mechanisms. Taken together, these implications emphasize that individual psychological capabilities should complement, rather than substitute for, sustainable organizational conditions. For banking organizations, addressing silent resignation may therefore require a combination of stronger work–life boundary protection, thoughtful development of emotional capabilities, and empowerment practices supported by meaningful opportunities for employee influence.

### 6.3 Limitations

Several methodological and contextual limitations should be acknowledged when interpreting the findings of this study. First, the cross-sectional nature of the data precludes definitive causal claims regarding the temporal ordering and directional dynamics of the examined relationships. Accordingly, the findings should be interpreted as associations rather than evidence of causal effects. In addition, although procedural remedies were implemented and collinearity diagnostics (inner VIF < 1.30) did not indicate substantial collinearity-related common method bias, all variables were assessed through self-report measures at a single measurement point. Common method variance therefore cannot be completely ruled out.

A further limitation concerns the operationalization of work–life balance. Work–life balance was operationalized using the reverse-scored work-to-family conflict dimension of Netemeyer et al. (1996). Although this approach captures the extent to which work demands interfere with employees’ family and non-work lives, it does not encompass the full multidimensional nature of work–life balance, including life-to-work processes and potentially positive forms of work–life interaction. Furthermore, although emotional intelligence significantly moderated the relationship between work–life balance and silent resignation, the interaction effect size was small (*f*^2^ = 0.015). The moderation finding should therefore be interpreted as a statistically significant but modest boundary condition rather than evidence of a large practical difference across levels of emotional intelligence.

Finally, the contextual specificity of the sample should be considered. The participants were exclusively white-collar employees of a commercial bank in Türkiye, where regulatory compliance, audit intensity, and operational standardization are pronounced. Generalizing the observed relationships to more decentralized, creative, flexible, or institutionally different work environments therefore requires empirical caution.

### 6.4 Recommendations for future research

Future research should build on the present findings by employing designs capable of addressing the temporal and measurement limitations of this study. Multi-wave longitudinal, daily-diary, and experience-sampling methodologies could capture fluctuations in work–life conditions and silent resignation over time and provide stronger evidence regarding temporal ordering and potential directional dynamics. Such designs could examine whether changes in work–life conditions precede changes in silent resignation or whether reciprocal relationships emerge over time. Subsequent studies could also incorporate multi-source evaluations, including supervisor ratings, peer assessments, and objective behavioral indicators such as recorded overtime or participation in discretionary organizational activities. Combining these indicators with employee self-reports may reduce reliance on a single source and provide a broader assessment of withdrawal-related behavior.

Future investigations should also adopt more comprehensive operationalizations of work–life balance. Multidimensional instruments capturing both work-to-life and life-to-work directions, together with positive processes such as work–life enrichment and facilitation, could provide a more complete understanding of how different dimensions of the work–life interface are associated with silent resignation. Replicating the conceptual model across industries characterized by different degrees of structural autonomy, flexibility, and regulatory oversight would also provide a stronger empirical test of the contextual boundaries identified in the present study, particularly with respect to psychological empowerment. Cross-cultural comparisons could further determine whether institutional and cultural contexts shape the relationships among work–life conditions, individual psychological characteristics, and silent resignation.

Finally, future research could examine emotional intelligence and psychological empowerment as mediators, antecedents, or components of more complex conditional process models rather than exclusively as moderators. Theoretically related mechanisms such as psychological contract fulfillment or breach, perceived organizational support, job autonomy, and burnout could also be incorporated to examine how work–life experiences are translated into silent resignation. Such alternative structural configurations may help clarify the psychological and exchange-based mechanisms underlying employees’ decisions to restrict discretionary contributions.

## Data Availability

The raw data supporting the conclusions of this article are not publicly available due to confidentiality commitments made to the participants during the informed consent process. Requests for further information may be directed to the corresponding author.
